# antiSMASH 4.0—improvements in chemistry prediction and gene cluster boundary identification

**DOI:** 10.1093/nar/gkx319

**Published:** 2017-04-28

**Authors:** Kai Blin, Thomas Wolf, Marc G. Chevrette, Xiaowen Lu, Christopher J. Schwalen, Satria A. Kautsar, Hernando G. Suarez Duran, Emmanuel L. C. de los Santos, Hyun Uk Kim, Mariana Nave, Jeroen S. Dickschat, Douglas A. Mitchell, Ekaterina Shelest, Rainer Breitling, Eriko Takano, Sang Yup Lee, Tilmann Weber, Marnix H. Medema

**Affiliations:** 1 Novo Nordisk Foundation Center for Biosustainability, Technical University of Denmark, 2800 Kgs. Lyngby, Denmark; 2 Leibniz Institute for Natural Product Research and Infection Biology—Hans-Knöll-Institute, 07745 Jena, Germany; 3 Laboratory of Genetics, University of Wisconsin—Madison, Madison, WI 53706, USA; 4 Bioinformatics Group, Wageningen University, 6708PB Wageningen, Netherlands; 5 Department of Chemistry, University of Illinois at Urbana-Champaign, Urbana, IL 61801, USA; 6 Warwick Integrative Synthetic Biology Centre, University of Warwick, Coventry CV4 7AL, UK; 7 Department of Chemical and Biomolecular Engineering & BioInformatics Research Center, Korea Advanced Institute of Science and Technology, Daejeon 34141, South Korea; 8 Faculty of Sciences, University of Lisbon, 1749-016 Lisbon, Portugal; 9 Kekulé-Institute of Organic Chemistry and Biochemistry, University of Bonn, 53121 Bonn, Germany; 10 Carl R. Woese Institute for Genomic Biology, University of Illinois at Urbana-Champaign, Urbana, IL 61801, USA; 11 Manchester Synthetic Biology Research Centre (SYNBIOCHEM), Manchester Institute of Biotechnology, University of Manchester, Manchester M1 7DN, UK

## Abstract

Many antibiotics, chemotherapeutics, crop protection agents and food preservatives originate from molecules produced by bacteria, fungi or plants. In recent years, genome mining methodologies have been widely adopted to identify and characterize the biosynthetic gene clusters encoding the production of such compounds. Since 2011, the ‘antibiotics and secondary metabolite analysis shell—antiSMASH’ has assisted researchers in efficiently performing this, both as a web server and a standalone tool. Here, we present the thoroughly updated antiSMASH version 4, which adds several novel features, including prediction of gene cluster boundaries using the ClusterFinder method or the newly integrated CASSIS algorithm, improved substrate specificity prediction for non-ribosomal peptide synthetase adenylation domains based on the new SANDPUMA algorithm, improved predictions for terpene and ribosomally synthesized and post-translationally modified peptides cluster products, reporting of sequence similarity to proteins encoded in experimentally characterized gene clusters on a per-protein basis and a domain-level alignment tool for comparative analysis of *trans*-AT polyketide synthase assembly line architectures. Additionally, several usability features have been updated and improved. Together, these improvements make antiSMASH up-to-date with the latest developments in natural product research and will further facilitate computational genome mining for the discovery of novel bioactive molecules.

## INTRODUCTION

Natural products, also referred to as secondary or specialized metabolites, are the basis of many drugs and are also important molecules for agricultural and nutritional applications; moreover, they play key roles in scientific research as chemical probes to study many aspects of molecular and cellular biology. The observation that the genomes of many microorganisms contain multiple biosynthetic gene clusters (BGCs) that code for the production of such molecules has led to a paradigm shift in natural products research: within the last 10 years, genome mining has been established as an important technology complementing the bioassay- and chemistry-driven classical natural products discovery process (1). This fundamental change was supported by the development and public availability of various genome mining software tools that are usable by wet-lab microbiologists and chemists (as reviewed in (2–4)), such as NP.searcher (5), antiSMASH (6–8), NaPDoS (9) and recently PRISM/GNP (10,11).

The comprehensive open-source BGC mining platform antiSMASH (6–8) was first released in 2011 and has been regularly updated with extended functionality. antiSMASH facilitates the mining of bacterial and fungal genomes and is tightly interconnected with plantiSMASH, a new variant for BGC mining in plants (12), the antiSMASH database (13) and the Minimum Information on Biosynthetic Gene Cluster (MIBiG) repository of experimentally characterized BGCs (14).

Here, we report version 4 of antiSMASH, which includes several major extensions, such as gene cluster boundary prediction for fungal BGCs, improved chemistry predictions for terpene, ribosomal peptide and non-ribosomal peptide BGCs, comparative alignment of *trans*-AT polyketide synthase (PKS) assembly lines and TTA codon annotation. Moreover, an improved user interface was introduced, along with several other usability and efficiency improvements. The public antiSMASH web server is freely accessible at http://antismash.secondarymetabolites.org.

### NEW FEATURES AND UPDATES

#### Improved prediction of gene cluster boundaries

**Figure 1.**
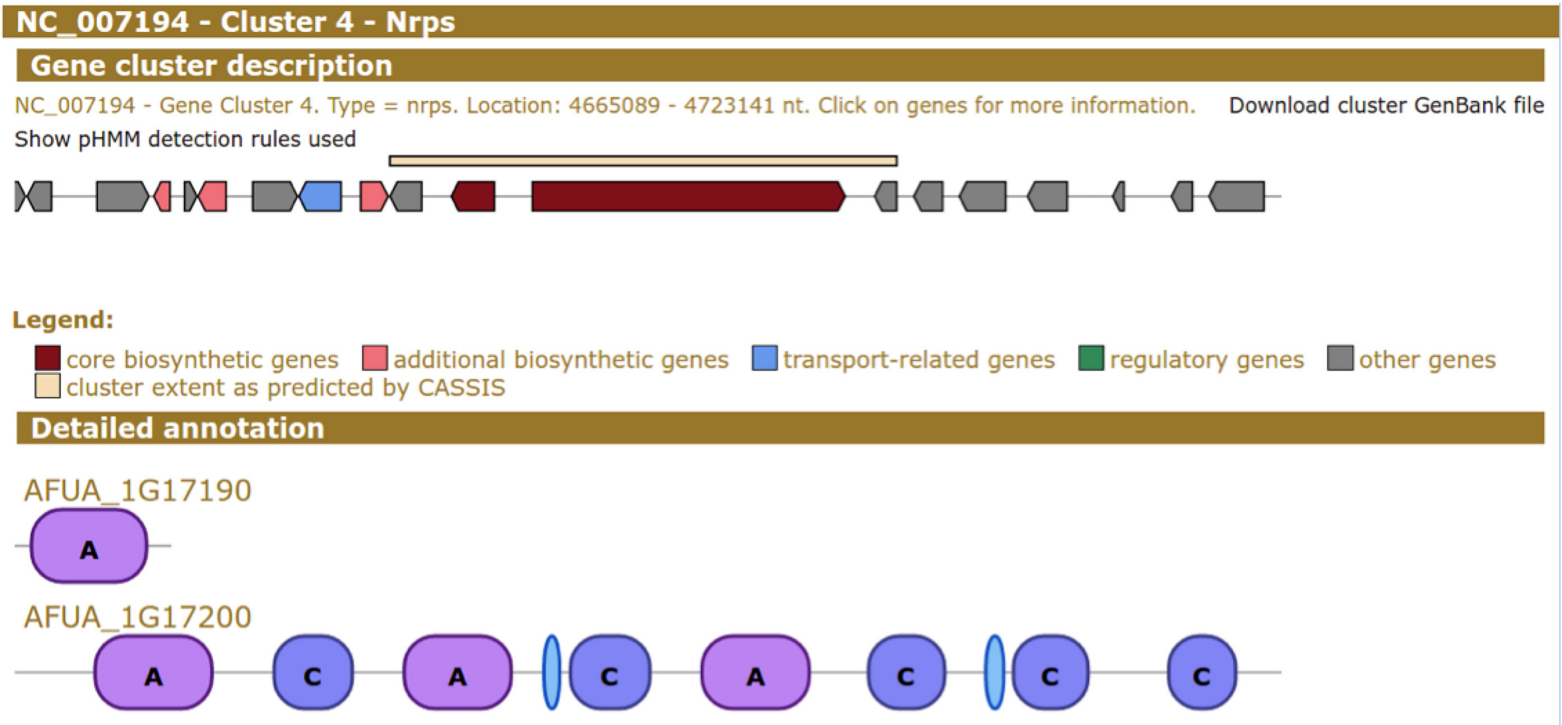
Gene cluster border prediction by the Cluster Assignment by Islands of Sites (CASSIS) algorithm. The fourth cluster on chromosome 1 of *Aspergillus nidulans* is shown. The cream-colored bar above the gene arrows spans the genes predicted to be clustered by CASSIS. Further genes in the surrounding are displayed for additional context. Similar functionality is available when using ClusterFinder to predict gene cluster borders.

Estimating the boundaries of BGCs is a continuing challenge for genome mining tools. Traditionally, antiSMASH has opted for a ‘greedy’ approach by design, in order to ensure a greater likelihood of including all pertinent biosynthetic genes. The rationale behind this was that expert users would be better at estimating cluster boundaries than automated algorithms would. However, for certain purposes, it is still highly beneficial for users to review a computer-assisted estimate of where a BGC may start and end. For this reason, antiSMASH has now added two methods to predict the boundaries of BGCs. For fungal genomes, the Cluster Assignment by Islands of Sites (CASSIS) algorithm (15) is used for this purpose, which identifies genes within the BGC that share a common pathway-specific regulatory motif (Figure 1). Additionally, for both bacterial and fungal genomes, the user can now choose to use the ClusterFinder algorithm (16) to estimate cluster boundaries based on frequencies of locally encoded protein domains detected by Pfam (17) (based on these being either more or less BGC-like). If the user selects one of the BGC boundary prediction options (ClusterFinder for bacteria and fungi, CASSIS for fungi only), the extents of the predicted cluster region are displayed as bars above the BGC and also annotated in the GenBank files that can be downloaded.

#### New algorithms for non-ribosomal peptide and terpene chemistry prediction

Since the first version of antiSMASH, three algorithms have been used within the pipeline to predict the substrate specificities of non-ribosomal peptide synthetase (NRPS) adenylation (A) domains: the support-vector machine (SVM) and active-site motif (ASM) prediction methods from NRPSPredictor2 (18) and the profile HMM (pHMM)-based method from Minowa *et al*. Since then, several new algorithms have been published to predict A-domain specificity (19–21). More recently, Chevrette *et al*. (manuscript in review) substantially expanded the training sets for these algorithms, introduced an additional (phylogenetics-based) algorithm (PrediCAT), benchmarked all algorithms systematically and constructed an ensemble prediction method (called SANDPUMA) that outperformed each method individually. To benefit from the latest insights in this field, we have now replaced the previous prediction algorithms with the SANDPUMA predictions; these provide not only the ensemble outputs, but also the individual outputs of the underlying SVM, ASM, PrediCAT and pHMM algorithms. Since the benchmark comparison had shown the Minowa method (22) to be the least reliable of all previously published methods, this algorithm was judged to be uninformative and has been removed from the antiSMASH pipeline.

In addition to the prediction of non-ribosomal peptide chemistry, antiSMASH now also provides chemical structure predictions for the products of bacterial terpene synthases (23). To this end, a terpene cyclase-specific version of PrediCAT (see Supplementary Figure S1 and Table S1) has been included, to predict terpene cyclization patterns (such as 1,6-, 1,10- or 1,11 cyclizations) based on phylogenetic relationships with known enzymes from a documented reference set of terpene cyclases: when a query enzyme forms a monophyletic clade with enzymes with a known cyclization chemistry, this cyclization pattern is assigned to the query as a prediction. These predictions (see Supplementary Figure S1 for accuracy assessment) are then reported alongside the name of and sequence identity to the most closely related experimentally characterized homolog. It should be noted that the predictions are only performed for those terpene BGCs that encode mono-, sesqui- or diterpene cyclases (Pfams PF01397 and/or PF03936) and not for those that (only) encode phytoene synthases, tetraterpene cyclases, oxidosqualene cyclases, tryptophan dimethylallyltransferases, geranylgeranyl diphosphate (GGPP) synthases and/or lycopene cyclases.

#### Improved RiPP BGC identification and structure prediction

Ribosomally synthesized and Post-translationally modified Peptides (RiPPs) constitute a growing area of natural products research. antiSMASH supports researchers in predicting 15 distinct classes of RiPP BGCs. Previously, antiSMASH predicted only lanthipeptide precursors using a relatively limited pHMM-based approach. The current version of antiSMASH now provides a more sophisticated prediction and classification for class I lanthipeptides as well as lasso peptides, sactipeptides and thiopeptides. Given that RiPPs start as gene-encoded precursor peptides prior to post-translational modification, amino acid sequence prediction provides a wealth of information regarding the structure of the final product. However, the open-reading frames (ORFs) encoding these peptides are often overlooked by automated analysis and can be highly sequence variable, necessitating the need for current precursor identification methods.

To assist in identifying the precursor peptide-encoding gene, antiSMASH now utilizes the algorithm from the genome-mining platform Rapid ORF Description and Evaluation Online (RODEO) (24), which uses a combination of heuristic scoring, SVM and motif analysis to evaluate all candidate precursor peptides in a putative RiPP BGC. To broaden its applicability, the RODEO algorithm was extended to perform precursor prediction not only for lasso peptides, but also for thiopeptides, class I lanthipeptides and sactipeptides (see Supplementary Text 1 and Figures S2–4). When submitting an annotated nucleotide sequence to antiSMASH, the algorithm evaluates small genes that are already part of this annotation, as well as all other small ORFs in intergenic regions across the predicted cluster, in order to mitigate issues with gene prediction.

For the RiPP classes analyzed by the RODEO algorithm, antiSMASH reports: (i) the respective class of RiPP (e.g. lasso peptide or thiopeptide, etc.), (ii) a predicted leader peptide cleavage site and (iii) any potential C-terminal proteolytic processing. Given the post-translational simplicity of lasso peptides, a molecular mass is also calculated, accounting for the number of disulfide bridges. For thiopeptides, the macrocycle size and potential amidation are predicted as well. Molecular weight predictions are not given for the other RiPP subclasses owing to their extensive and variable post-translational modifications.

#### *Trans*-AT PKS domain alignments

**Figure 2.**
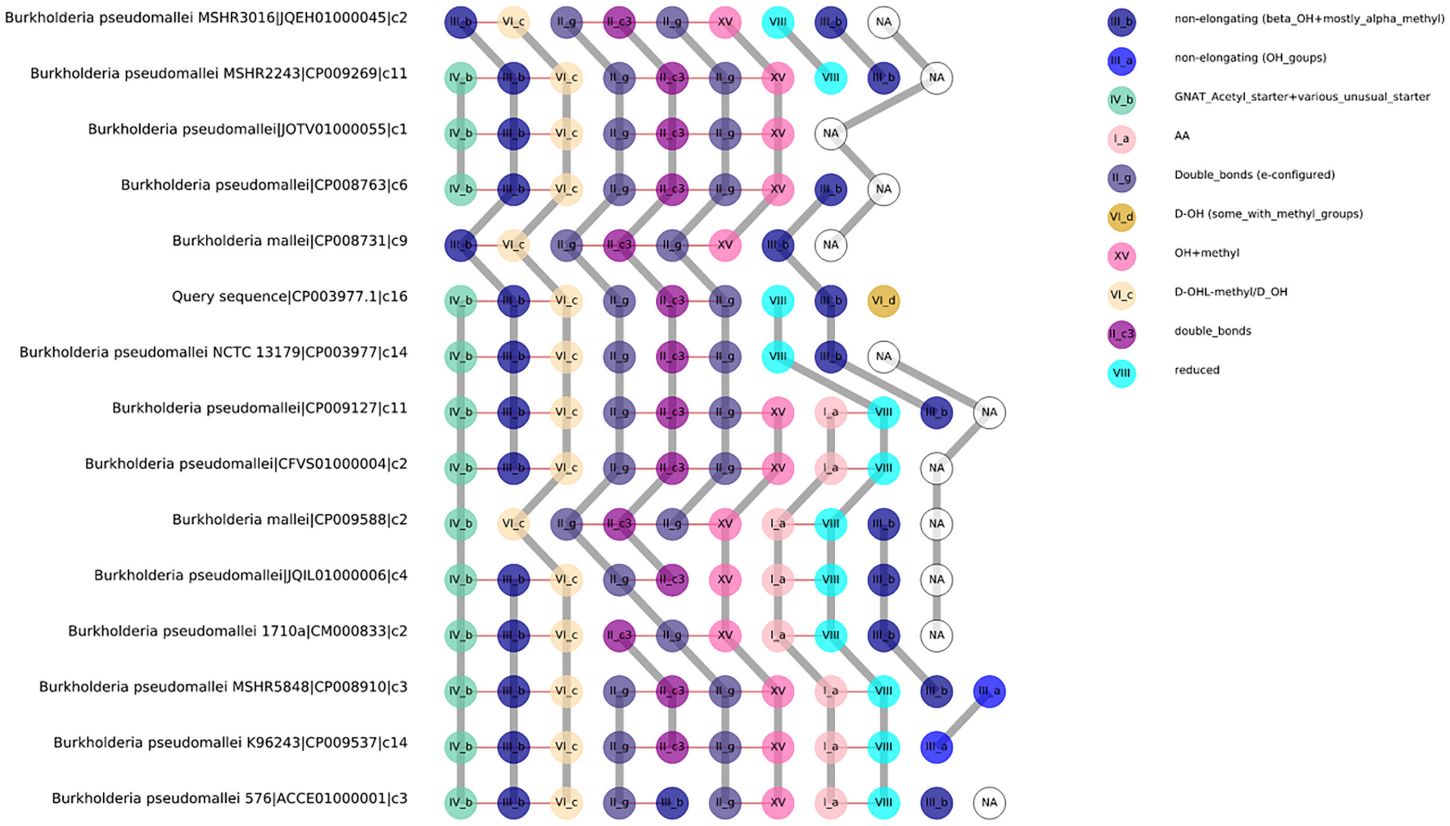
Visualization of *trans*-AT PKS assembly-line alignments. The top 15 most closely related assembly lines are visualized together with the query sequence (which represents the identified BGC currently in view). When clicking on a domain, its location (amino acid coordinates) within the parent protein are displayed and clicking on the gray connecting edges will trigger a display of the sequence identity between homologous domains based on a MAFFT multiple sequence alignment.

Several key classes of natural products are produced by multimodular enzymatic assembly lines. Standard similarity searches (as performed in antiSMASH's ClusterBlast module) do not reveal major insights between the natural product structures and the genes for the corresponding multidomain proteins that encode their biosynthetic enzymes. In order to better address this issue, we have now included an assembly line alignment method for *trans*-AT PKS (E. Helfrich, X. Lu *et al*. manuscript in preparation), which uses reference phylogenies of ketosynthase (KS) domains to assign KS domains from identified gene clusters into clades that correspond to a certain type of polyketide chemistry. Based on this classification, the encoded assembly line is then aligned to reference assembly lines from known BGCs in MIBiG (14) based on a distance metric that involves the Jaccard index, Goodman–Kruskal gamma function and domain duplication index of KS domain clades at empirically determined weights of 0.5, 0.25 and 0.25, respectively (see also (25)). The assembly lines that are most closely related to the query are then selected and clustered using Unweighted Pair Group Method with arithmetic mean clustering with the same metric and displayed in a visual alignment, in which each KS domain clade is annotated with a distinct color and a text description of the associated chemistry (Figure 2). This analysis allows for a rapid assessment of biochemical relationships between the products of these assembly lines, in order to identify new variants of known molecules or to find novel polyketide scaffolds.

#### TTA codon annotation

*Streptomyces* and related genera are important producers of clinically used antibiotics, such as tetracyclines or erythromycin, or drugs to treat parasitic worms such as avermectin. These bacteria have GC-contents of >70% and thus a skew toward higher GC triplets in their codon usage. While genes involved in primary metabolism almost exclusively use CTC codons to code for Leu, key genes in secondary metabolism and cell differentiation often contain TTA codons. As the expression of the TTA-codon specific Leu-tRNA-gene *bldA* is tightly controlled and the Leu-tRNA only accumulates in later stages of growth, this offers an additional level of regulation (26–28). The expression of the BGCs therefore does not only require activation at the transcriptional level, but also the presence of the TTA-specific Leu-tRNA. This must be considered, for example, for heterologous BGC expression in other streptomycete hosts or metabolic engineering approaches. Therefore, a new feature was included in antiSMASH version 4 to automatically scan all BGCs for the presence of TTA codons and annotate these in the graphical cluster overview and the GenBank/EMBL result files.

#### Usability and efficiency improvements

antiSMASH comes with an updated, larger ClusterBlast database for comparative gene cluster analysis. In order to keep the runtime of the ClusterBlast analysis at acceptable levels with the much larger database, antiSMASH now uses the BLAST-compatible DIAMOND algorithm (29) to calculate results for ClusterBlast (against all ±220,000 BGCs currently detected in NCBI GenBank) and KnownClusterBlast (against experimentally characterized BGCs from MIBiG (14). ClusterBlast results are now cross-referenced to the antiSMASH database (13), whenever present there, through hyperlinks on the matched clusters; this allows researchers to quickly get a more complete view of these BGCs. Also, for each gene in a predicted gene cluster, an individual BLAST search is now automatically run against all proteins encoded in BGCs deposited in MIBiG (14); this helps researchers to predict functions of individual genes based on similarity of their encoded amino acid sequence to those of experimentally characterized proteins, even when the rest of the surrounding gene clusters are not similar.

In order to simplify selecting the correct input settings, separate submission pages were created for fungal sequences (http://fungismash.secondarymetabolites.org/) and plant sequences (http://plantismash.secondarymetabolites.org/). The main antiSMASH website is now focused on bacterial and archaeal sequences. The metabolic modeling functionality along with an EC number prediction option that were introduced in antiSMASH version 3 were removed again, as they led to extremely long run times and high server load. An updated version with improved reaction rules for secondary metabolite biosynthetic pathways will be released as a separate, but still closely linked program.

In addition to GenBank- and EMBL-formatted files, gene annotations can now also be added to FASTA sequences by also uploading a GFF3-formatted file. To assist job submission and retrieval from third-party tools running upstream or downstream analyses such as the CRISPR single guide RNA finding tool CRISPy-web (30) or the Antibiotics Resistance Target Seeker service (31), the antiSMASH web component now supports a REST-like (32) web API.

### CONCLUSIONS AND FUTURE PERSPECTIVES

With the new features now introduced (Table 1), the antiSMASH framework continues to improve through the concerted action of researchers in the natural products community. A number of additional features are still in development, including application of the visual assembly line alignments to NRPSs, detailed gene cluster boundary prediction through phylogenetic profiling and detection of putative resistance genes inside BGCs.

**Table 1.** Overview of analyzes integrated into antiSMASH

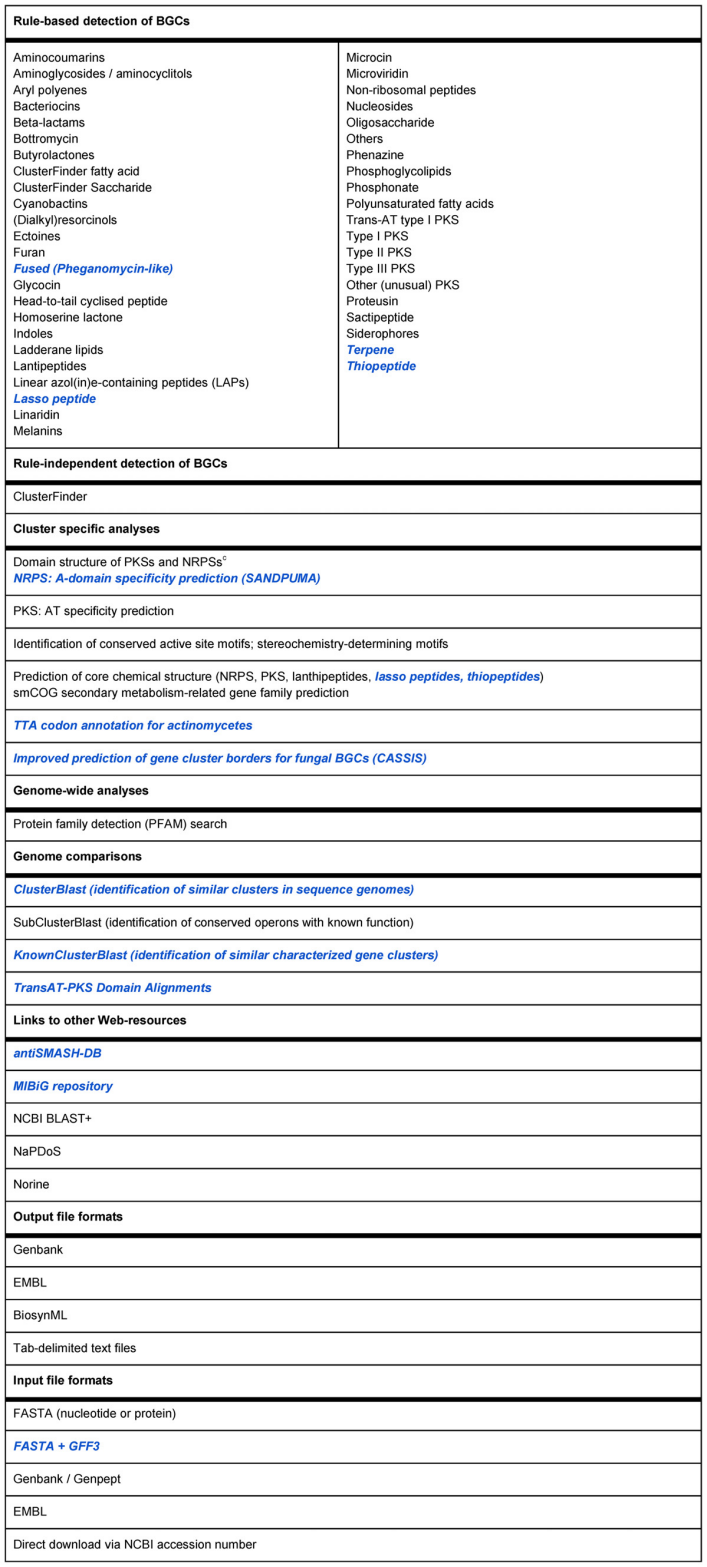

With regard to chemistry prediction of the products of NRPSs and PKSs, we have opted to be conservative for the moment. The recently introduced PRISM pipeline (11) does a great job of automatically predicting a wide range of possible products of each BGC, which facilitates automated matching to large-scale metabolomic data. However, the majority of antiSMASH users still rely on manual comparison of BGCs with smaller-scale experimental data; we feel that this approach benefits more from reliable predictions of substructures and substrate specificities (and refraining from making lower-confidence combinatorial predictions). In this respect, PRISM and antiSMASH offer complementary functionalities and the user can opt to use either pipeline based on the intended research purposes.

We continue to strive for interoperability with other services. For example, antiSMASH predictions are also available through the Joint Genome Institute's IMG-ABC (33) as well as Genoscope's framework MicroScope (34); connections to EFI-EST (35) and other tools are being investigated. Also, we remain committed to collaborating with other researchers worldwide and invite expert feedback as well as technical contributions from the community to improve this important piece of software.

## AVAILABILITY

antiSMASH is available from http://antismash.secondarymetabolites.org/. This website is free and open to all users and there is no login requirement. Source code is available from https://bitbucket.org/antismash/antismash/.

## Supplementary Material

Supplementary Data
