# Supplementary material for: antiSMASH 4.0—improvements in chemistry prediction and gene cluster boundary identification
Source: Nucleic Acids Res. 2017 Apr 28;45(Web Server issue):W36–41. doi: 10.1093/nar/gkx319 (PMC5570095; doi:10.1093/nar/gkx319)
Supplement: Supplementary Data [file gkx319_Supp.zip › nar-00613-web-b-2017-File004.docx]

**Supplementary Data**

**antiSMASH 4.0 — Improvements in Chemistry Prediction and Gene Cluster Boundary Identification**

Kai Blin^1^, Thomas Wolf^2^, Marc G. Chevrette^3^, Xiaowen Lu^4^, Christopher J. Schwalen^5^, Satria A. Kautsar^4^, Hernando G. Suarez Duran^4^, Emmanuel L.C. de los Santos^6^, Hyun Uk Kim^1,7^, Mariana Nave^8^, Jeroen S. Dickschat^9^, Douglas A. Mitchell^5,10^, Ekaterina Shelest^2^, Rainer Breitling^11^, Eriko Takano^11^, Sang Yup Lee^1,7^, Tilmann Weber^1^*, and Marnix H. Medema^4^*

^1^ Novo Nordisk Foundation Center for Biosustainability, Technical University of Denmark, Kgs. Lyngby, DK. ^2^ Leibniz Institute for Natural Product Research and Infection Biology – Hans-Knöll-Institute, Jena, DE. ^3^ Laboratory of Genetics, University of Wisconsin - Madison, Madison, US. ^4^ Bioinformatics Group, Wageningen University, Wageningen, NL. ^5^ Department of Chemistry, University of Illinois at Urbana-Champaign, Urbana, IL, USA. ^6^ Warwick Integrative Synthetic Biology Centre, University of Warwick, Coventry, UK. ^7^ Department of Chemical and Biomolecular Engineering & BioInformatics Research Center, Korea Advanced Institute of Science and Technology, Daejeon, KR. ^8^ Faculty of Sciences, University of Lisbon, Lisbon, PT. ^9^ Kekulé-Institute of Organic Chemistry and Biochemistry, University of Bonn, Bonn, Germany. ^10^ Department of Microbiology, Carl R. Woese Institute for Genomic Biology, University of Illinois at Urbana-Champaign, Urbana, IL, USA.^11^ Manchester Synthetic Biology Research Centre (SYNBIOCHEM), Manchester Institute of Biotechnology, University of Manchester, UK

* Corresponding authors

Email addresses: KB: [kblin@biosutain.dtu.dk](mailto:kblin@biosutain.dtu.dk), TWo: [thomas.wolf@leibniz-hki.de](mailto:thomas.wolf@leibniz-hki.de), MGC: [chevrette@wisc.edu](mailto:chevrette@wisc.edu), XL:[luxiaowen0205@gmail.com](mailto:luxiaowen0205@gmail.com), CS: [schwale2@illinois.edu](mailto:schwale2@illinois.edu), SK: [satria.kautsar@wur.nl](mailto:satria.kautsar@wur.nl), HSD: [hernando.suarezduran@wur.nl](mailto:hernando.suarezduran@wur.nl), EdLS: [E.De-Los-Santos@warwick.ac.uk](mailto:E.De-Los-Santos@warwick.ac.uk), HUK: [ehukim@kaist.ac.kr](mailto:ehukim@kaist.ac.kr), MN: [mariana.nave@gmail.com](mailto:mariana.nave@gmail.com), DM: [douglasm@illinois.edu](mailto:douglasm@illinois.edu), ES: [ekaterina.shelest@leibniz-hki.de](mailto:ekaterina.shelest@leibniz-hki.de), RB: [rainer.breitling@manchester.ac.uk](mailto:rainer.breitling@manchester.ac.uk), ET: [eriko.takano@manchester.ac.uk](mailto:eriko.takano@manchester.ac.uk), SYL: [leesy@kaist.ac.kr](mailto:leesy@kaist.ac.kr), TWe: [tiwe@biosustain.dtu.dk](mailto:tiwe@biosustain.dtu.dk), MHM: [marnix.medema@wur.nl](mailto:marnix.medema@wur.nl)

**Contents**

Supplementary Figure 1. Terpene PrediCAT 2

Supplementary Table 1 PrediCAT Training Set 3

Supplementary Text: RODEO algorithm. 3

Supplementary Figure 2. Histogram of test set for lasso peptides. 4

Supplementary Figure 3. Histogram of test set for sactipeptides. 7

Supplementary Figure 4. Histogram of test set for thiopeptides. 10

Supplementary Figure 5. Histogram of test set for class I lanthipeptides. 13

Supplementary Figure 6. Heatmaps plotting score optimization. 16

**
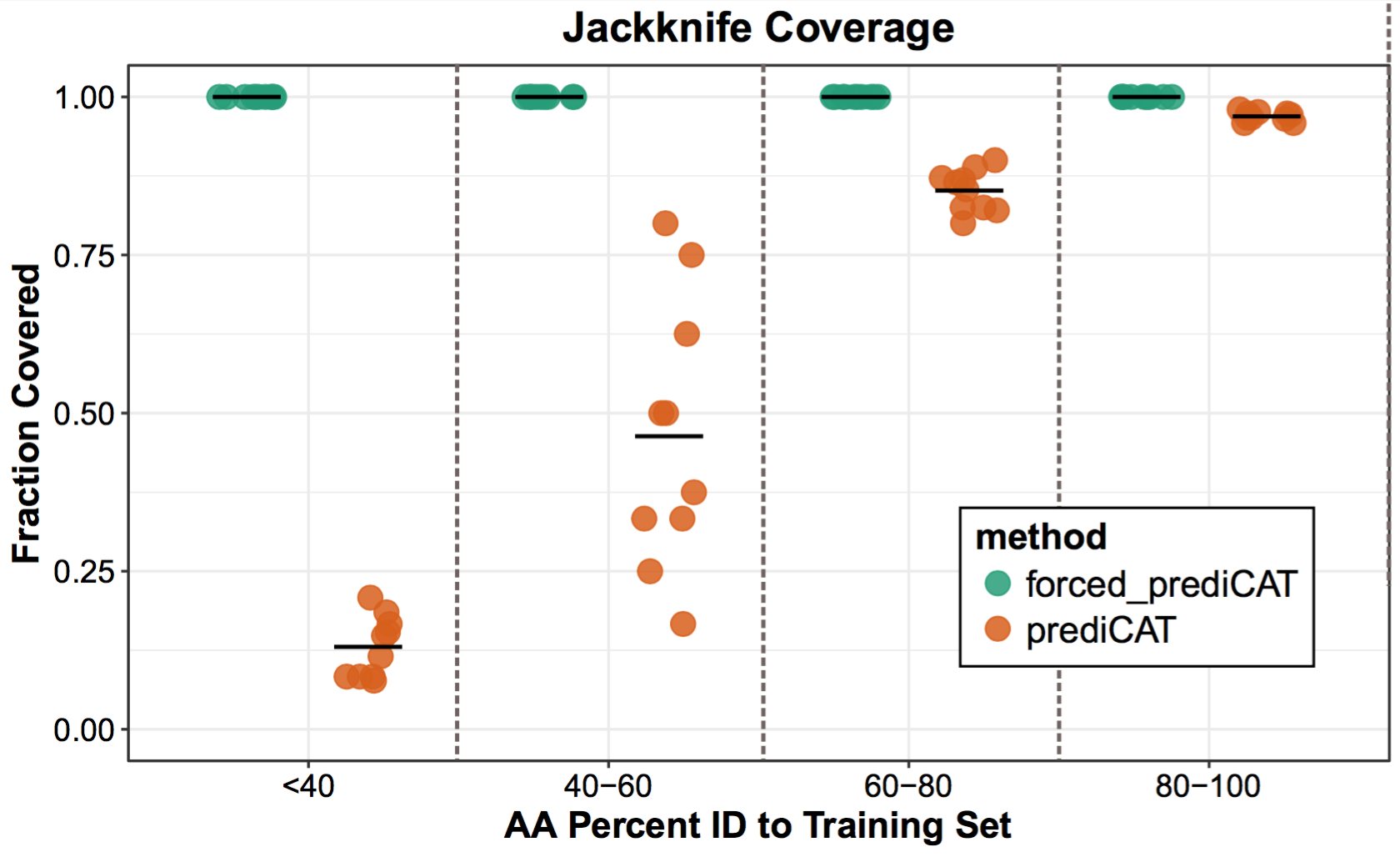

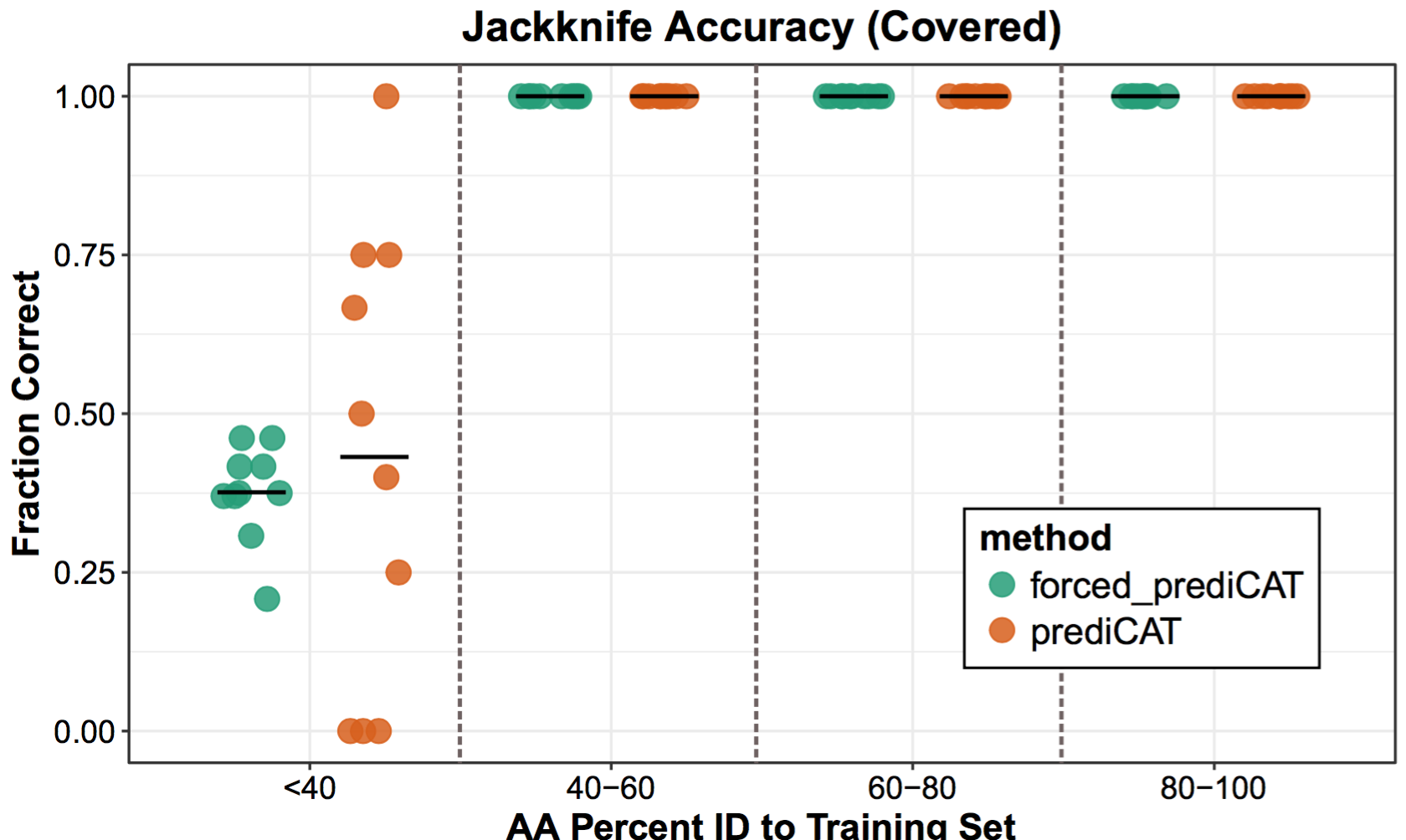
**Supplementary Figure 1: Terpene PrediCAT.

Two types of predictions are made by PrediCAT, forced or unforced. In the default (unforced) mode that is used in antiSMASH, PrediCAT only returns predictions when there is a clear monophyletic relationship with a member of the training set in a reconstructed phylogenetic tree. The upper panel reports accuracy of predictions made according to a ten-fold jackknife validation, the lower panel reports the fraction of inputs for which a prediction is made.

# Supplementary Table 1 PrediCAT Training Set

(see Excel file)**:** Training set used for terpene PrediCAT. Columns indicate functional annotation, cyclization pattern, protein accession, taxonomy of parent strain, and topology.

# Supplementary Text: RODEO algorithm.

Scoring was performed analogously to methods described previously (Tietz et al., in press, doi: 10.1038/nchembio.2319). Briefly, for RiPP precursor scoring and identification, the six-frame translated peptides in the local genomic context of the BGC was analyzed using a set of heuristic parameters, motif analysis (via MEME/FIMO) and support vector machines (SVM). Heuristics were determined by inspection of BGC and precursor characteristics of bona fide RiPPs from these classes. Weight and scoring values for the heuristics were adjusted manually to yield higher-scoring values for the expected positive cases. Examples of these precursors were further analyzed by the MEME motif suite for identification of sequence motifs where noted. Lastly, the support vector classifier was trained using the noted parameters on a subset of RODEO-derived BGCs. Hyperparameters (kernel, class weight, C and gamma) were optimized using iterative training with either 5- or 10-fold cross-validation. Optimization was evaluated by analysis of precision, recall, F1 and scoring (precision x recall) metrics to select the best hyperparameter values. These hyperparameters were then used in the classifier function on a reserved set distinct from the initial training set and inspected manually for plausibility.

|  | **Lasso peptides** | **Lanthipeptides (class I)** | **Sactipeptides** | **Thiopeptides** |
| --- | --- | --- | --- | --- |
| Number of examples in training set | 2495 | 1497 | 1082 | 1503 |
| Number of SVM features | 170 | 152 | 149 | 126 |
| Optimized Precision | 0.969 | 0.910 | 0.913 | 0.950 |
| Optimized Recall | 0.958 | 0.925 | 0.975 | 0.952 |
| Score | 0.928 | 0.842 | 0.890 | 0.904 |
| Optimized C | 2.83E+05 | 9.77E+06 | 9.77E+06 | 2.83E+05 |
| Optimized gamma | 1.00E-08 | 1.78E-09 | 1.00E-09 | 1.00E-09 |


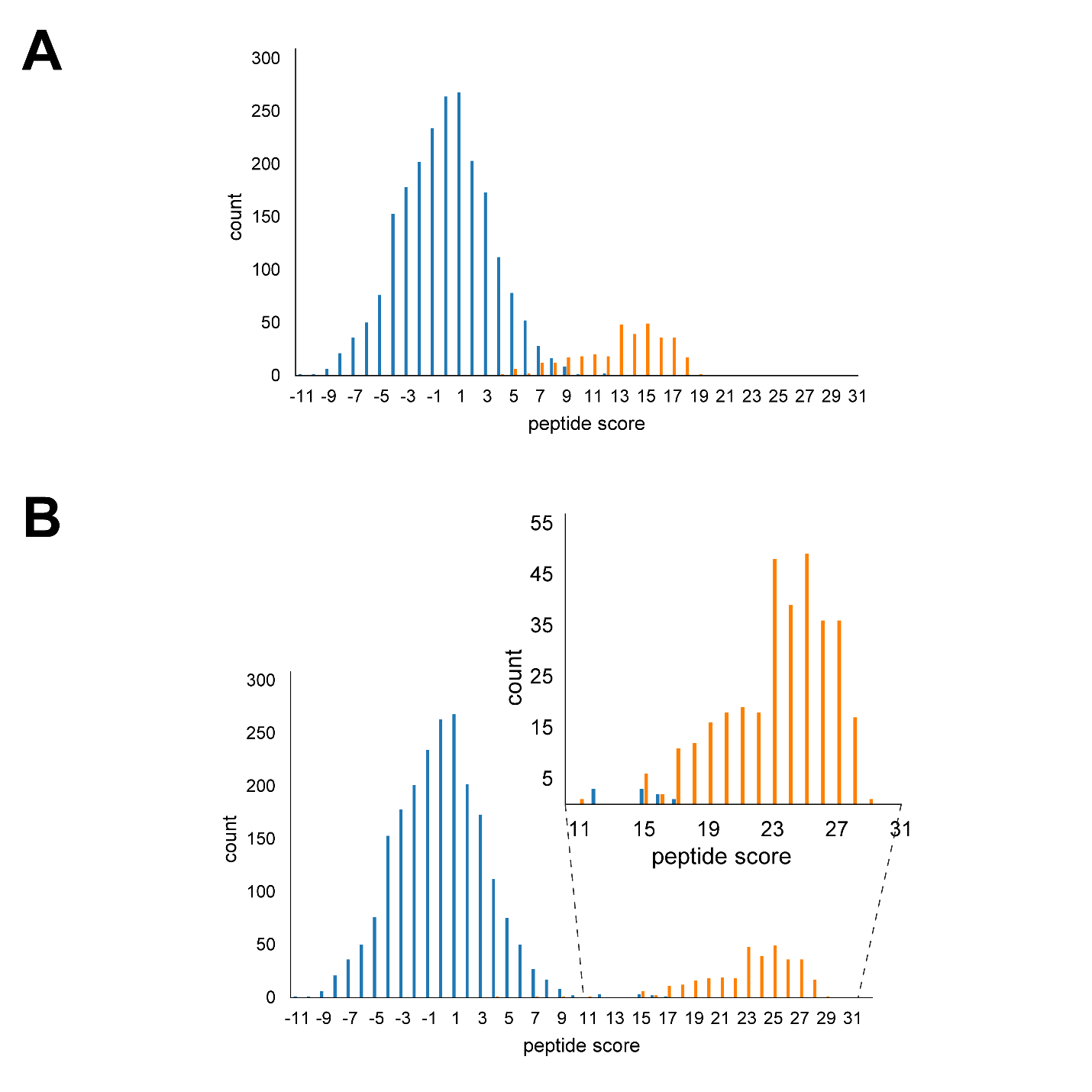


# Supplementary Figure 2. Histogram of test set for lasso peptides.

(data adapted from Tietz et al., in press, doi: 10.1038/nchembio.2319) Distinguishing between non-valid sactipeptide precursors (blue) and valid sactipeptide precursors (orange) was evaluated by separation of histogram populations as a function of peptide scoring. **A.** Plot of peptide counts with scoring determined by heuristics alone. **B.** Plot of peptide counts with scoring determined by heuristics, motif analysis and SVM.

**Lasso peptide heuristic scoring metrics***

|  | **Feature** | **Weight** |
| --- | --- | --- |
| **Heuristic scoring** | Precursor is within 500 nucleotides of any biosynthetic protein (E, B, C) | +1 |
|  | Precursor is within 150 nucleotides of any biosynthetic protein (E, B, C) | +1 |
|  | Greater than 1000 nucleotides from every biosynthetic protein (E, B, C) | -2 |
|  | Core region has 2 or 4 Cys residues | +1 |
|  | Leader region is longer than core region | +2 |
|  | Core has 7 (Glu) or 8(Glu/Asp) or 9 (Asp) membered ring possible | +1 |
|  | Core starts with G | +2 |
|  | Leader region contains GxxxxxT | +3 |
|  | Peptide and lasso cyclase are on same strand | +1 |
|  | Leader/core region length ratio < 2 and > 0.5 | +1 |
|  | Core starts with Cys and has an even number of Cys | 0 |
|  | Core contains no Gly | -4 |
|  | Core has at least one aromatic residue | +1 |
|  | Core has at least 2 aromatic residues | +2 |
|  | Core has odd number of Cys | -2 |
|  | Leader region contains Trp | -1 |
|  | Leader region contains Lys | +1 |
|  | Leader region has Cys | -2 |
|  | Gene cluster does not contain PF13471 | -2 |
| **+MEME** | Peptide contains sequence motif #2 | +2 |
|  | Peptide contains ANY lasso sequence motif | +2 |
|  | Peptide contains NO lasso sequence motifs | -1 |
|  | Peptide utilizes alternate start codon | -1 |
| **+SVM** | SVM classifies as valid | +10 |

*Final threshold: 15*

**Lasso peptide SVM parameters***

| **Feature** | **Data type** | **Feature** | **Data type** |
| --- | --- | --- | --- |
| Calcd. lasso peptide mass (Da) | Float | Core has adjacent identical aas | Boolean |
| Minimum distance from C, E, or B protein (nt) | Integer | Core length (aa) | Integer |
| Within 500 nt of C, E, or B | Boolean | Leader length (aa) | Integer |
| Within 150 nt of C, E, or B | Boolean | Precursor length (aa) | Integer |
| Further than 1000 nt from C, E, or B | Boolean | Leader/core ratio | Float |
| Core has 2 or 4 Cys | Boolean | Number of Pro in first 9 aa of core | Integer |
| Leader is longer than core | Boolean | Estimated core charge at neutral pH | Integer |
| Can form most common size ring | Boolean | Estimated leader charge at neutral pH | Integer |
| Leader has GxxxxxT motif? | Boolean | Estimated precursor charge at neutral pH | Integer |
| Core starts with G? | Boolean | Absolute value of core charge at neutral pH | Integer |
| Core and BGC in same direction? | Boolean | Absolute value of leader charge at neutral pH | Integer |
| Ratio leader/core < 2 and > 0.5 | Boolean | Absolute value of precursor charge at neutral pH | Integer |
| Core starts with Cys and has even number of Cys | Boolean | Number in leader of each amino acid | Integer |
| No Gly in core | Boolean | Number in leader of each amino acid type (aromatic, aliphatic, hydroxyl, basic, acidic) | Integer |
| Core has at least 1 aromatic aa | Boolean | Number in core of each amino acid | Integer |
| Core has at least 2 aromatic aa | Boolean | Number in core of each amino acid type (aromatic, aliphatic, hydroxyl, basic, acidic) | Integer |
| Core has odd number of Cys | Boolean | Number in entire precursor of each amino acid | Integer |
| Leader has Trp | Boolean | Number in entire precursor of each amino acid type (aromatic, aliphatic, hydroxyl, basic, acidic) | Integer |
| Leader has Lys | Boolean | Presence of each of motifs 1–16 | Boolean |
| Leader has Cys | Boolean | Total motifs hit | Integer |
| Cluster has PF00733 | Boolean | MEME/FIMO score of each of motifs 1–16 | Float |
| Cluster has PF05402 | Boolean | Sum of MEME scores | Float |
| Cluster has PF13471 | Boolean | No motifs present | Boolean |
| Leader has LxxxxxT motif | Boolean | Alternate start codon used | Boolean |
| Core starts with aa (for each) | Boolean |  |  |

*Scoring matrix for heuristics and SVM training parameters adapted from Tietz et al., in press, doi: 10.1038/nchembio.2319

Charge is calculated by the sum of residues predicted to have a charge at a neutral pH. Lys/Arg are taken to be +1 formal charge each and Asp/Glu are taken to be -1 formal charge each. The net charge is defined as the sum of positive and negative charges.


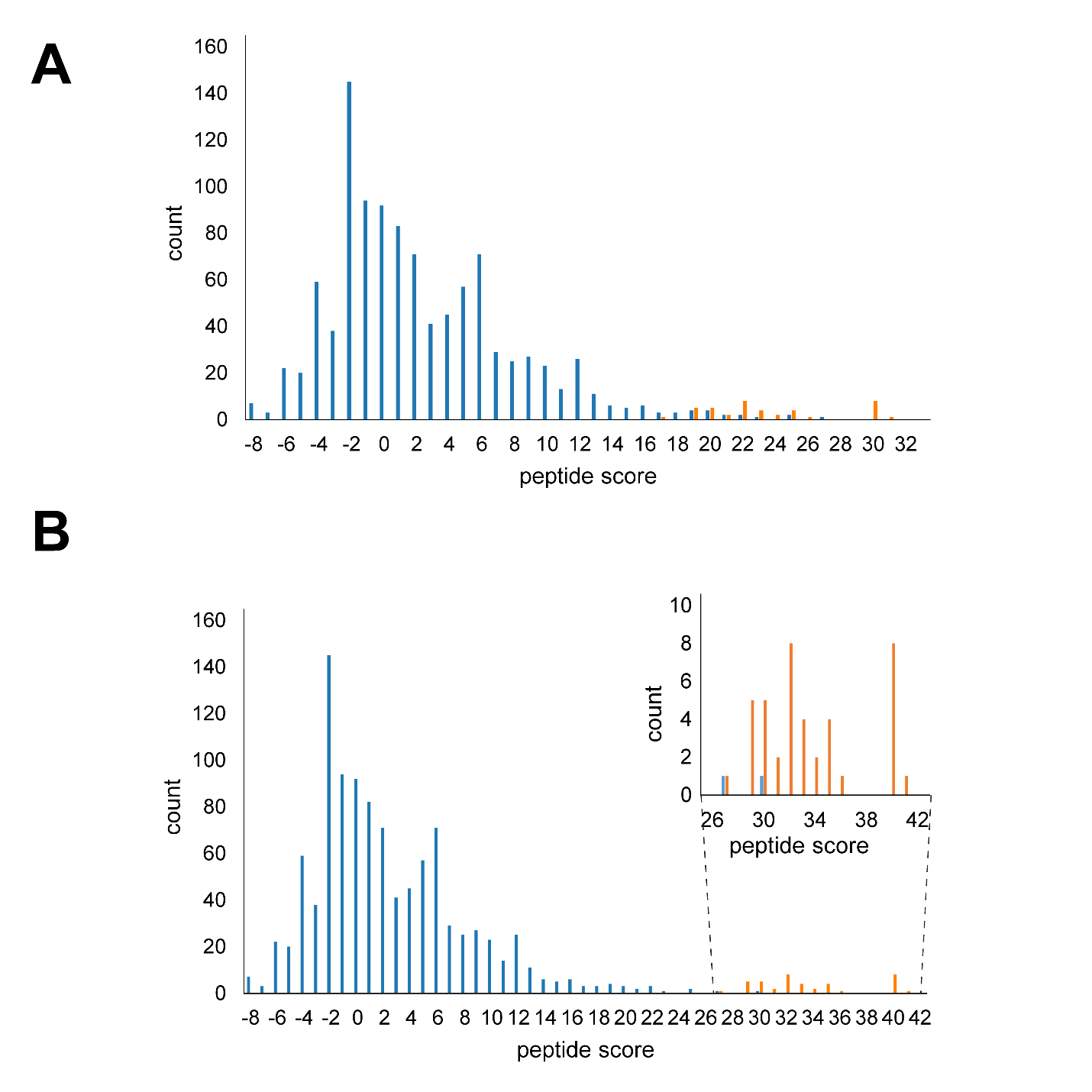


# Supplementary Figure 3. Histogram of test set for sactipeptides.

Distinguishing between non-valid sactipeptide precursors (blue) and valid sactipeptide precursors (orange) was evaluated by separation of histogram populations as a function of peptide scoring. **A.** Plot of peptide counts with scoring determined by heuristics alone. **B.** Plot of peptide counts with scoring determined by heuristics, motif analysis and SVM.

**Sactipeptide heuristic scoring metrics**

|  | **Feature** | **Weight** |
| --- | --- | --- |
| **Heuristic scoring** | Precursor is within 500 nt of rSAM? | +1 |
|  | Precursor is within 150 nt of rSAM? | +1 |
|  | Precursor is further than 1000 nt of rSAM? | -2 |
|  | Ratio of length of precursor N-term to 1st Cys to length of full precursor is >0.25 and < 0.60 | +2 |
|  | Ratio of length of precursor N-term to 1st Cys to length of full precursor is <0.25 or > 0.60 | -2 |
|  | Contains three or more Cys | +4 |
|  | Contains less than 3 Cys | -4 |
|  | Contains motif(s) CxC/Cx_2_C/Cx_3_C/Cx_5_C | (+1/+1/+1/+2) |
|  | Contains motif(s) CC/CCC | (-2/-2) |
|  | No Cys in last 1/4^th^ of precursor length | +1 |
|  | Cys in last 1/4^th^ of precursor length | -1 |
|  | 2 Cys in first 2/3rd of precursor length, 1 Cys in last 1/3rd of precursor length | +1 |
|  | Peptide hits SboA (PF11420) HMM | +3 |
|  | Peptide hits SkfA (TIGR04404) HMM | +3 |
|  | Peptide hits SCIFF (TIGR03973) HMM | +2 |
|  | Cluster has PqqD/RRE (PF05402) | +1 |
|  | Cluster has SPASM domain (PF13186) | +1 |
|  | PF04055 (rSAM) domain starting ≥ residue 80 | +1 |
|  | Cluster has peptidase (PF05193/PF00082/PF03572/PF00675) | (+1/+1/+1/+1) |
|  | Cluster has transporter (PF00005/PF00664) | (+1/+1) |
|  | Cluster has response regulator (PF00072) | +1 |
|  | Cluster has major facilitator (PF07690) | +1 |
|  | Cluster has ATPase (PF13304) | +1 |
|  | Cluster has Fer4_12 (PF13353) | +1 |
|  | Cluster has rSAM (PF04055) | +2 |
|  | Cluster has no recognized peptidase (PF05193/PF00082/PF03572/PF00675) | -2 |
|  | C-terminal portion (ratio of length from last Cx_n_C to C-terminus to length of precursor) is < 0.35 or > 0.65 | -2 |
|  | C-terminal portion (ratio of length from last Cx_n_C to C-terminus to length of precursor) is > 0.35 and < 0.65 | +3 |
|  | Sactipeptide ring profile sum > 1 | +2 |
| **+SVM** | SVM classifies as valid | +10 |

*Final threshold: 26*

**Sactipeptide SVM parameters**

| **Feature** | **Data type** | **Feature** | **Data type** |
| --- | --- | --- | --- |
| Heuristic sactipeptide features (see previous) | Boolean | Number in leader of each amino acid | Integer |
| Length of leader peptide | Integer | Number in leader of each amino acid type (aromatic, aliphatic, hydroxyl, basic, acidic) | Integer |
| Length of precursor peptide | Integer | Number in core of each amino acid | Integer |
| Length of core peptide | Integer | Number in core of each amino acid type (aromatic, aliphatic, hydroxyl, basic, acidic) | Integer |
| Length of core / length of precursor ratio | Float | Number in entire precursor of each amino acid | Integer |
| Length of core / length of leader ratio | Float | Number in entire precursor of each amino acid type (aromatic, aliphatic, hydroxyl, basic, acidic) | Integer |
| Ratio of length of N-terminus to first Cys / length of core | Float | Number of each peptidase Pfam hit (PF05193/PF00082/PF03572/PF00675/PF02517/PF02163/PF00326) | Integer |
| Number of occurrences of Cx_N_C motifs | Integer | Number of each ABC transporter Pfam hit (PF00005/PF00664) | Integer |
| Average distance between Cx_N_C motifs | Float | Number of each response regulator Pfam hit (PF00072) | Integer |
| Ratio of length from last Cx_N_C to C-terminus / length of core | Float | Number of each major facilitator Pfam hit (PF07690) | Integer |
| Number of instances of Cx_N_C where N = 1 | Integer | Number of each ATPase Pfam hit (PF02518/PF13304) | Integer |
| Number of instances of Cx_N_C where N = 2 | Integer | Number of each Fer4_12 Pfam hit (PF13353) | Integer |
| Number of instances of Cx_N_C where N = 3 | Integer | Number of each rSAM Pfam hit (PF04055) | Integer |
| Number of instances of Cx_N_C where N = 4 | Integer |  |  |
| Number of instances of Cx_N_C where N = 5 | Integer |  |  |
| Number of instances of Cx_N_C where N = 6 | Integer |  |  |
| Length of rSAM including PqqD domain | Integer |  |  |
|  |  |  |  |
|  |  |  |  |

**Red** = Not included in the heuristic Boolean scoring

Charge is calculated by the sum of residues predicted to have a charge at a neutral pH. Lys/Arg are taken to be +1 formal charge each and Asp/Glu are taken to be -1 formal charge each. The net charge is defined as the sum of positive and negative charges.


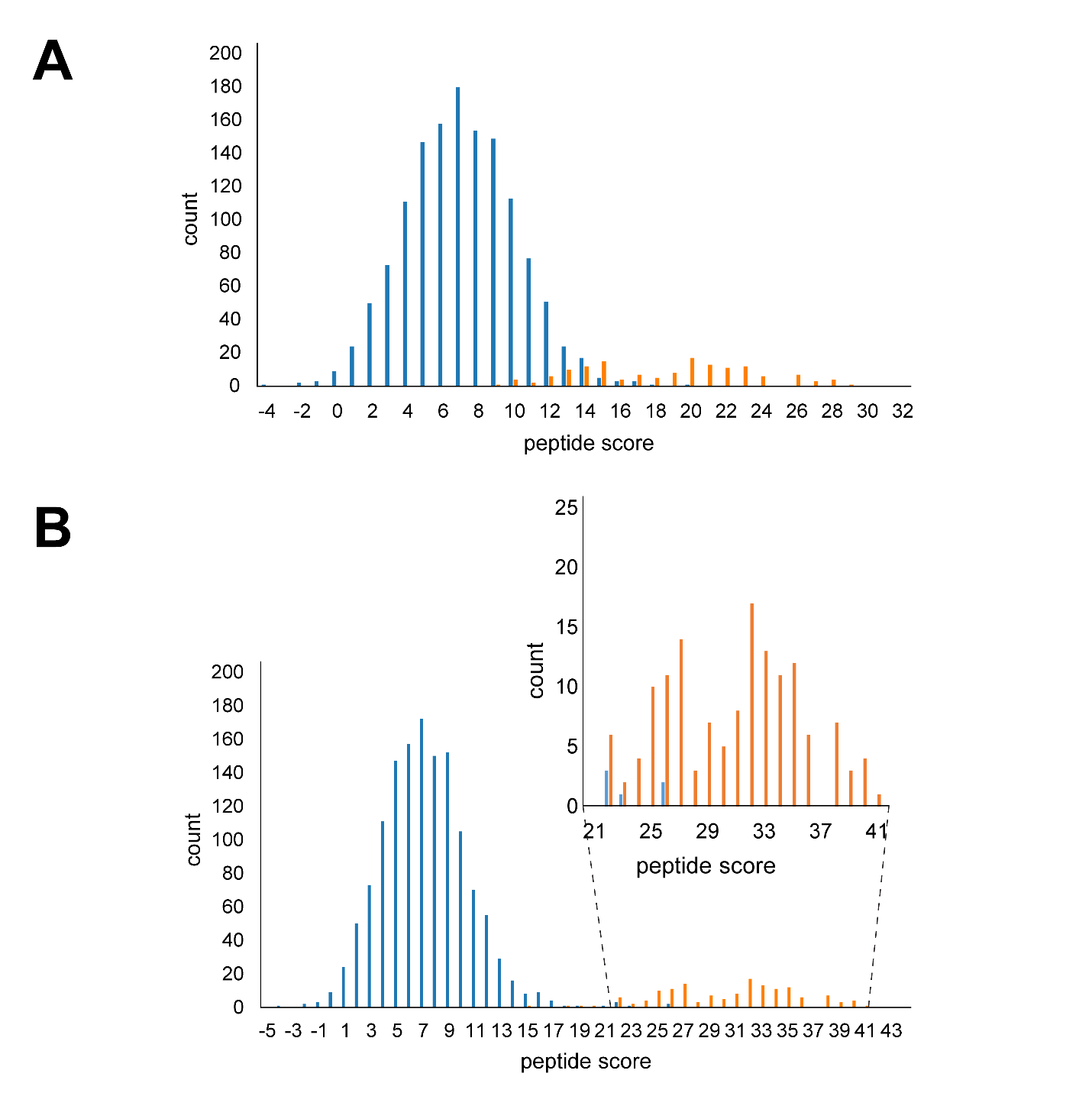


# Supplementary Figure 4. Histogram of test set for thiopeptides.

Distinguishing between non-valid thiopeptide precursors (blue) and valid thiopeptide precursors (orange) was evaluated by separation of histogram populations as a function of peptide scoring. **A.** Plot of peptide counts with scoring determined by heuristics alone. **B.** Plot of peptide counts with scoring determined by heuristics, motif analysis and SVM.

**Thiopeptide heuristic scoring metrics**

|  | **Feature** | **Weight** |
| --- | --- | --- |
| **Heuristic scoring** | Cluster contains TOMM YcaO (PF02624) | +2 |
|  | Cluster contains LanB N-terminal domain (PF04738) | +2 |
|  | Cluster contains LanB C-terminal domain (PF14028) | +2 |
|  | Cluster contains TOMM dehydrogenase (PF00881) | +2 |
|  | Cluster contains rSAM methyltransferase (PF04055) | +2 |
|  | Cluster contains P450 (PF00067) | +1 |
|  | Cluster contains ABC transporter (PF00005) | +1 |
|  | Cluster contains ABC transporter (PF01061) | +1 |
|  | Cluster contains ABC transporter (PF12698) | +1 |
|  | Cluster contains abhydrolase1 (PF12697) | +1 |
|  | Cluster contains abhydrolase2 (PF00561) | +1 |
|  | Precursor peptide contains CSS/CTT motif | +(1,1) |
|  | Precursor peptide contains SS/SSS/SSSS motif | +(1,1,2) |
|  | Precursor peptide contains CC/CCC/CCCC motif | +(1,1,2) |
|  | Precursor peptide contains TT/TTT/TTTT motif | +(1,1,2) |
|  | Core peptide contains no Cys residues | -2 |
|  | Core peptide contains no Ser residues | -2 |
|  | Core peptide contains no Thr residues | -2 |
|  | Mass of core peptide (unmodified) < 2100 | +1 |
|  | Sum of repeating Cys/Ser/Thr > 4 | +2 |
|  | Average heterocyclizable residues (Cys/Ser/Thr) block length > 3 | +2 |
|  | Leader peptide number of negatively charged residues – number positively charged residues > 5 | +2 |
|  | Leader net charge > 0 | -2 |
|  | Leader contains a Cys? | -1 |
|  | Precursor peptide terminates in Cys/Ser/Thr | +1 |
|  | Core peptide contains ≥ 2 positively charged residues | -1 |
|  | Number of heterocyclizable residues (Cys/Ser/Thr) to total number of residues in precursor peptide ratio > 0.4 | +2 |
| **+SVM** | SVM classifies as valid | +10 |

*Final threshold: 20*

**Thiopeptide SVM parameters**

| **Feature** | **Data type** | **Feature** | **Data type** |
| --- | --- | --- | --- |
| Thiopeptide heuristic features (see previous) | Boolean | Number in leader of each amino acid | Integer |
| Number repeating blocks of Cys/Ser/Thr residues in core | Integer | Number in leader of each amino acid type (aromatic, aliphatic, hydroxyl, basic, acidic) | Integer |
| Number of core repeating Cys | Integer | Number in core of each amino acid | Integer |
| Number of core repeating Ser | Integer | Number in core of each amino acid type (aromatic, aliphatic, hydroxyl, basic, acidic) | Integer |
| Number of core repeating Thr | Integer | Number in entire precursor of each amino acid | Integer |
| Number of blocks of Cys/Ser/Thr residues in core | Integer | Number in entire precursor of each amino acid type (aromatic, aliphatic, hydroxyl, basic, acidic) | Integer |
| Average core Cys/Ser/Thr block length | Float |  |  |
| Unmodified precursor peptide mass | Float |  |  |
| Unmodified leader peptide mass | Float |  |  |
| Unmodified core peptide mass | Float |  |  |
| Length of Precursor | Integer |  |  |
| Length of Leader | Integer |  |  |
| Length of Core | Integer |  |  |
| Ratio of length of leader / length of core | Float |  |  |
| Ratio of number of Cys/Ser/Thr residues / length of core | Float |  |  |
|  |  |  |  |
|  |  |  |  |
|  |  |  |  |

Charge is calculated by the sum of residues predicted to have a charge at a neutral pH. Lys/Arg are taken to be +1 formal charge each and Asp/Glu are taken to be -1 formal charge each. The net charge is defined as the sum of positive and negative charges.


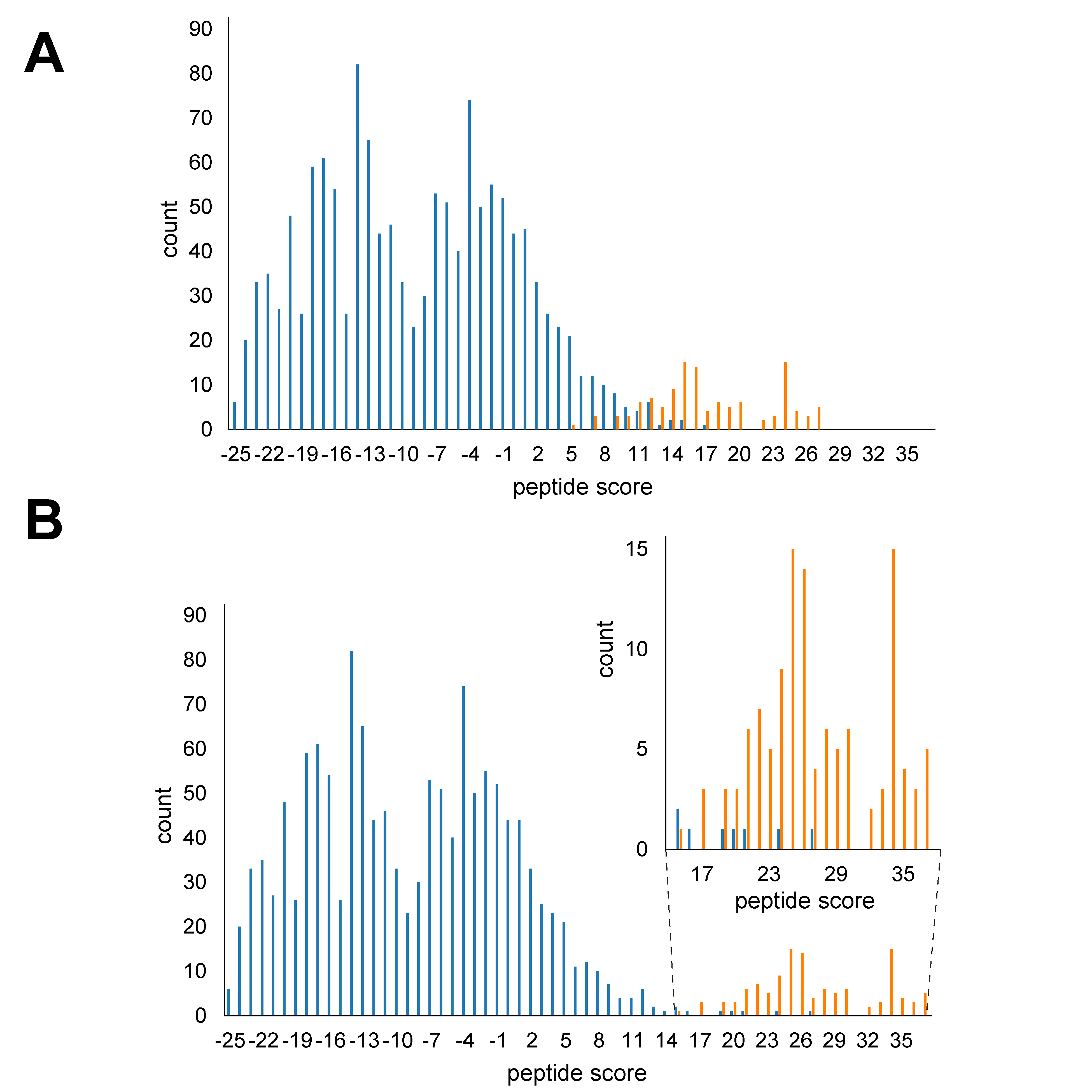


# Supplementary Figure 5. Histogram of test set for class I lanthipeptides.

Distinguishing between non-valid lanthipeptides precursors (blue) and valid lanthipeptides precursors (orange) was evaluated by separation of histogram populations as a function of peptide scoring. **A.** Plot of peptide counts with scoring determined by heuristics alone. **B.** Plot of peptide counts with scoring determined by heuristics, motif analysis and SVM.

**Lanthipeptide (class I) heuristic scoring metrics**

|  | **Feature** | **Weight** |
| --- | --- | --- |
| **Heuristic scoring** | Precursor is within 500 nt? | +1 |
|  | Cluster contains LanB dehydratase domain (PF04738) | +2 |
|  | Cluster contains Lan C cyclase domain (PF05147) | +2 |
|  | Cluster LACKS LanB dehydratase domain (PF04738) | -1 |
|  | Cluster LACKS Lan C cyclase domain (PF05147) | -1 |
|  | Cluster contains LanB dehydratase elimination C-terminal domain (PF14028) | +2 |
|  | Cluster contains S8 peptidase subtilase (PF00082) | +1 |
|  | Cluster contains C39 peptidase (PF03412) | +1 |
|  | Cluster contains ABC transporter (PF00005) | +1 |
|  | Cluster contains YcaO-like protein (PF02624) | -4 |
|  | Cluster contains ThiF-like protein (PF00899) | -4 |
|  | Precursor peptide mass < 4000 Da | -3 |
|  | Core peptide mass < 2000 Da | -3 |
|  | Precursor peptide hits gallidermin (PF02052) HMM* | +3 |
|  | Precursor peptide hits Pep5/epicidin-280 (PF0813) HMM* | +3 |
|  | Precursor peptide hits gallidermin superfamily (cl03420) HMM* | +3 |
|  | Precursor peptide hits lantibio_gallid (TIGR03731) HMM* | +3 |
|  | Precursor peptide hits lanti_SCO0268 superfamily (cl22812) HMM* | +3 |
|  | Precursor peptide hits LD_lanti_pre (TIGR04363) HMM* | +3 |
|  | Precursor peptide hits Antimicrobial18 (cl06940) HMM* | +3 |
|  | Precursor peptide hits Antimicrobial18 (PF08130) HMM* | +3 |
|  | Core peptide ≥ 35 residues | -2 |
|  | Core peptide contains CC motif (not in last 3 residues) | -3 |
|  | Core peptide contains C-terminal CC (within last 3 residues) | +2 |
|  | Leader peptide has > 4 negatively charge motifs | +1 |
|  | Leader peptide has net negative charge | +1 |
|  | Leader peptide contains FxLD motif | +2 |
|  | Core peptide contains DGCGxTC motif | +2 |
|  | Core peptide contains SFNS motif | +2 |
|  | Core peptide contains SxxLC motif | +2 |
|  | Core peptide contains CTxGC motif | +1 |
|  | Core peptide contains TPGC motif | +1 |
|  | Core peptide contains SFNSxC | +1 |
|  | Core peptide contains < 2 or < 3 Cys | (-3/-3) |
|  | No Cys/Ser/Thr in core peptide | (-10/-4/-4) |
|  | Lanthionine regex maximum ring number > 4 | +2 |
|  | Lanthionine regex maximum ring number < 3 | -2 |
|  | Lanthionine regex 4-membered ring (**greater than 2**)/5-membered ring (**greater than 2**)/6-membered ring (**greater than 0**)/7-membered ring (**greater than 0**)/8-membered ring (**greater than 0**) | (+2/+2/+2/+2/+1) |
| **+SVM** | SVM classifies as valid | +10 |

* Mutually exclusive: the +3 bonus is given once, if at least one of the HMMs hits to the precursor peptide.

*Final threshold: 14*

**Lanthipeptide (class I) SVM parameters**

| **Feature** | **Data type** | **Feature** | **Data type** |
| --- | --- | --- | --- |
| Heuristic lanthipeptide features (see previous) | Boolean | Number in leader of each amino acid | Integer |
| Precursor peptide mass (unmodified) | Float | Number in leader of each amino acid type (aromatic, aliphatic, hydroxyl, basic, acidic) | Integer |
| Leader peptide mass (unmodified) | Float | Number in core of each amino acid | Integer |
| Core peptide mass (unmodified) | Float | Number in core of each amino acid type (aromatic, aliphatic, hydroxyl, basic, acidic) | Integer |
| Leader residue position of FxLD motif | Integer | Number in entire precursor of each amino acid | Integer |
| Core residue position of Sx_4_C/Sx_5_C motif | Integer | Number in entire precursor of each amino acid type (aromatic, aliphatic, hydroxyl, basic, acidic) | Integer |
| Core residue position of Tx_4_C/Tx_5_C motif | Integer | log10 p-value MEME motif 1 | Float |
| Length of leader peptide | Integer | log10 p-value MEME motif 2 | Float |
| Length of core peptide | Integer | log10 p-value MEME motif 3 | Float |
| Length of precursor peptide | Integer | log10 p-value MEME motif 4 | Float |
| Ratio of length of leader peptide / length of core peptide | Float | log10 p-value MEME motif 5 | Float |
| Lanthionine regex maximum ring number | Integer |  |  |
| Lanthionine regex 4-membered ring count | Integer |  |  |
| Lanthionine regex 5-membered ring count | Integer |  |  |
| Lanthionine regex 6-membered ring count | Integer |  |  |
| Lanthionine regex 7-membered ring count | Integer |  |  |
| Lanthionine regex 8-membered ring count | Integer |  |  |
| Ratio of number of Cys in core peptide to sum of Ser/Thr in core peptide | Float |  |  |
| Ratio of number of Cys/Ser/Thr to length of core peptide | Float |  |  |
|  |  |  |  |

Charge is calculated by the sum of residues predicted to have a charge at a neutral pH. Lys/Arg are taken to be +1 formal charge each and Asp/Glu are taken to be -1 formal charge each. The net charge is defined as the sum of positive and negative charges.


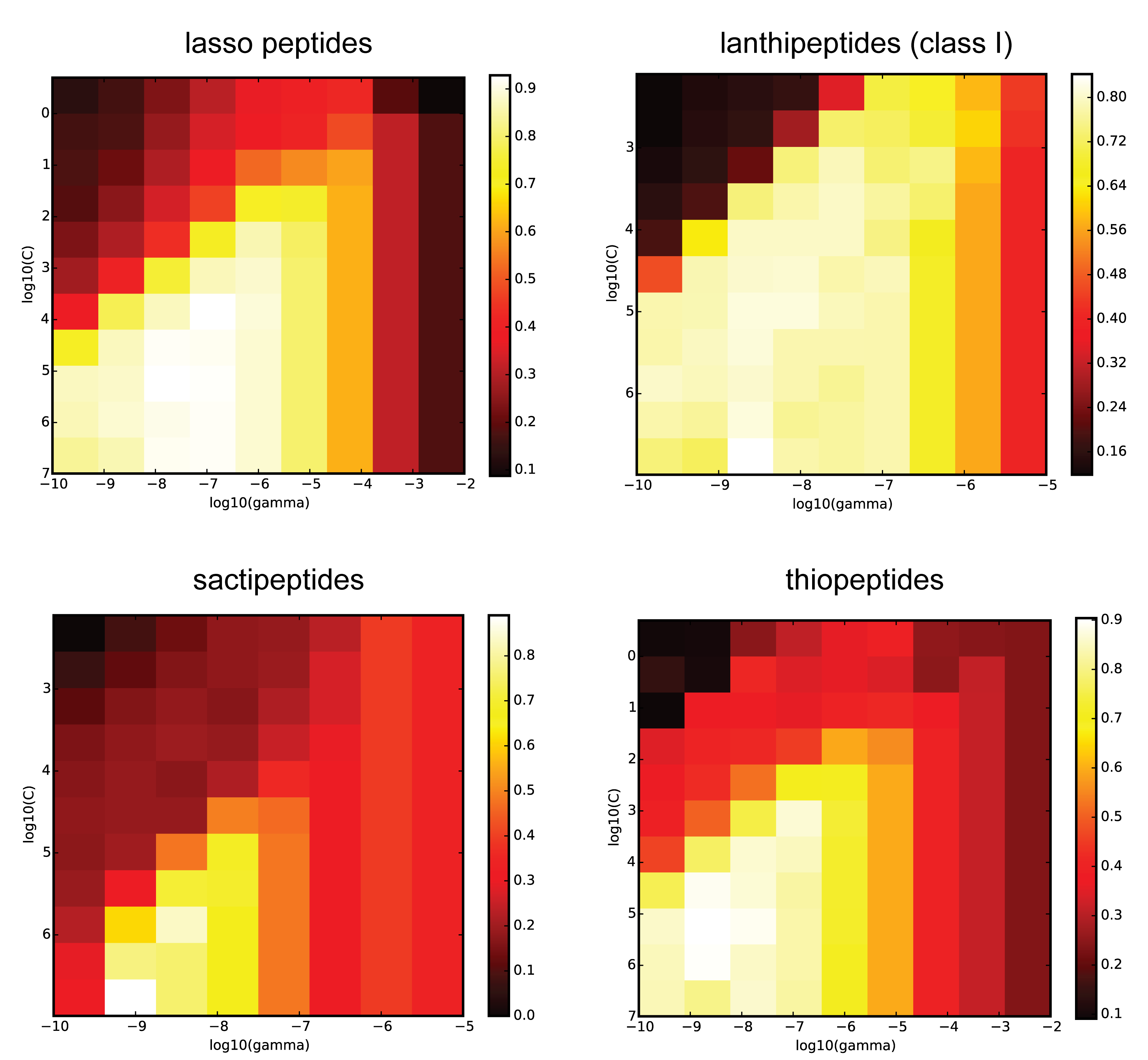


# Supplementary Figure 6. Heatmaps plotting score optimization.

Score values were determined as a function of c and gamma hyperparameters. Values of c and gamma were iterated and recall and precision were calculated (score was defined as recall × precision) over 5- and 10-fold cross validation.
